# Controversy of indomethacin eye drops in the treatment of rheumatoid arthritis-induced corneal ulceration: a case report

**DOI:** 10.1186/s13256-020-02600-9

**Published:** 2021-03-04

**Authors:** Simona Delia Nicoară, Ioana Damian

**Affiliations:** 1 Department of Ophthalmology, Iuliu Hațieganu University of Medicine and Pharmacy, 8, V. Babes Street, 400012 Cluj-Napoca, Romania; 2 Department of Ophthalmology, Emergency County Hospital, Cluj-Napoca, Romania

**Keywords:** Corneal perforation, Topical cyclosporine, Topical nonsteroidal anti-inflammatory eye drops, Rheumatoid arthritis

## Abstract

**Background:**

Perforation of the cornea is a rare finding in patients with rheumatoid arthritis (RA). Addressing a perforated cornea associated with RA is challenging, since its pathogenesis is not fully elucidated. Topical nonsteroidal anti-inflammatory drugs (NSAIDs) were developed to prevent cystoid macular edema following cataract surgery in patients at risk. Their prescription in inflammation of the anterior segment of the eye may induce negative effects on the ocular surface. We bring into focus a corneal perforation in a patient with RA who used indomethacin eye drops to treat corneal ulceration, but responded promptly to drug discontinuation and initiation of topical cyclosporine 0.1%. Our aim is to emphasize two issues: the contraindication of topical indomethacin in corneal defects, and the immediate positive impact of topical cyclosporine 0.1% on corneal healing.

**Case presentation:**

A 73-year-old Caucasian woman with a 13-year history of RA was treated for corneal ulceration in her oculus sinister (OS) with topical indomethacin and gentamicin. The patient was being treated with systemic immunosuppression and NSAIDs for the underlying RA and artificial tears in both eyes. No bandage contact lens was used. After 3 weeks of treatment, perforation of the left cornea occurred and the patient was referred to our hospital. Upon admission, visual acuity (VA) in the OS was 20/630. Slit lamp examination of the OS revealed paracentral corneal perforation, iris plugging the perforation site, shallow anterior chamber, clear aqueous humor, and clear lens. Anterior segment optical coherence tomography showed the inclavated iris in the perforation site and minimum corneal thickness of 101 µm. Topical NSAIDs were discontinued and topical treatment was initiated with tobramycin, tropicamide 1%, phenylephrine 10%, and artificial tears five times a day, and occlusive patch. For 5 days, there was no improvement, so topical cyclosporine 0.1% was started, one drop every evening. Within 7 days, the cornea had healed, the iris was liberated from the perforation site, the minimum corneal thickness increased to 250 µm, VA improved to 20/25, and the patient was free of symptoms.

**Conclusions:**

The main “takeaway” lessons from this case are that topical indomethacin should not be prescribed in cases of inflammation of the anterior segment of the eye, and that topical cyclosporine was efficacious in healing corneal perforation in our patient.

## Background

Perforation of the cornea is a rare finding in patients with rheumatoid arthritis (RA) [1, 2]. This paper reports the case of perforated corneal ulceration in a patient with RA that had been treated for 3 weeks with topical indomethacin prior to admission to our hospital. Although the pathogenesis of corneal perforation is complex [1, 2], we aim to call attention to the potentially devastating effects of topical indomethacin on the cornea, if not properly indicated [3, 4]. Topical nonsteroidal anti inflammatory drugs (NSAIDs) are designed to prevent cystoid macular edema after cataract surgery in patients at risk, not to treat inflammation of the anterior segment of the eye [3, 4]. Unfortunately, some eye care physicians prescribe them routinely for inflammation of the anterior segment of the eye, with potential side effects, among which corneal perforation is the most severe. We bring into focus a case of corneal perforation in a patient with RA with prolonged use of topical indomethacin, but who responded promptly to drug discontinuation and initiation of topical cyclosporine 0.1%. Our case joins a few other reports in the literature related to the occurrence of severe corneal lesions following prolonged topical treatment with NSAIDs, sounding a warning signal on their accurate indication [3, 4]. We also emphasize the immediate positive effect of topical cyclosporine 0.1% on the corneal healing process.

Informed consent was obtained from the patient regarding the publication of this case and the related images.

## Case presentation

A 73-year-old Caucasian woman with a 13-year history of RA complained of a marked decrease in visual acuity (VA) and intense pain in her oculus sinister (OS), which was red, accompanied by a sensation of hot leakage on her cheek, approximately 12 hours before admission to our hospital. For the past 3 weeks she had been treated in another service with gentamicin drops and indomethacin drops four times a day for a previously diagnosed corneal ulceration of the OS. No bandage contact lens was used. Before this episode, the patient did not have regular ophthalmological follow-ups. The patient had no history of hypertension or diabetes.

The patient was using methotrexate 2.5 mg three times a week for treatment of RA. For the dry eye syndrome, preservative-free lubricants were prescribed.

**Fig. 1:**
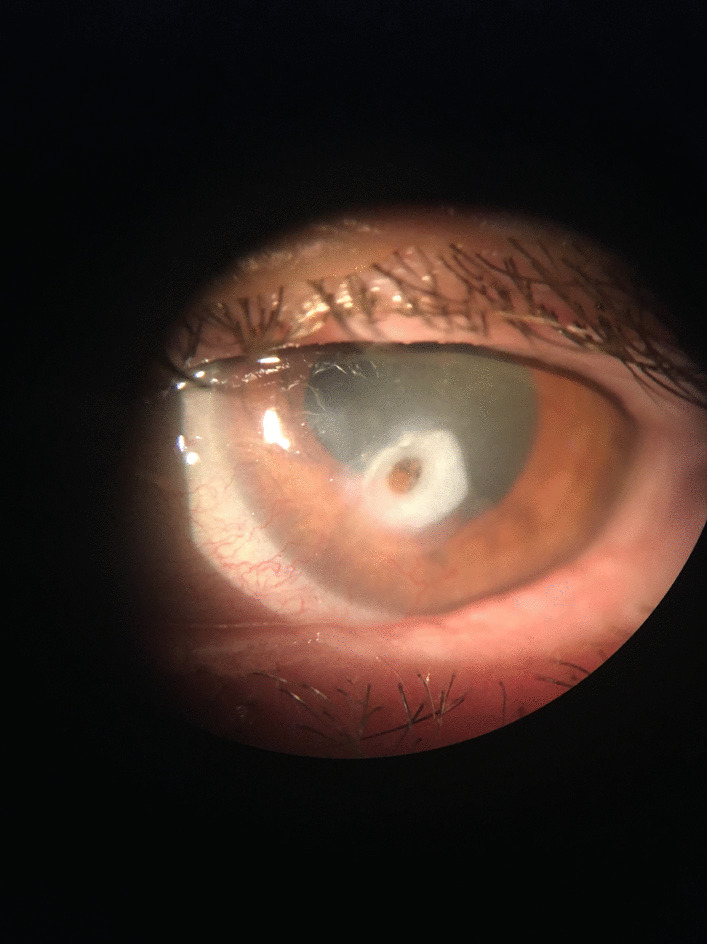
Slit lamp examination: perforated paracentral corneal ulceration, with dense perilesional infiltrate, stromal melting, a few corneal new vessels and stromal opacities, shallow anterior chamber, clear aqueous humor, iris plugging the corneal perforation

**Fig. 2:**
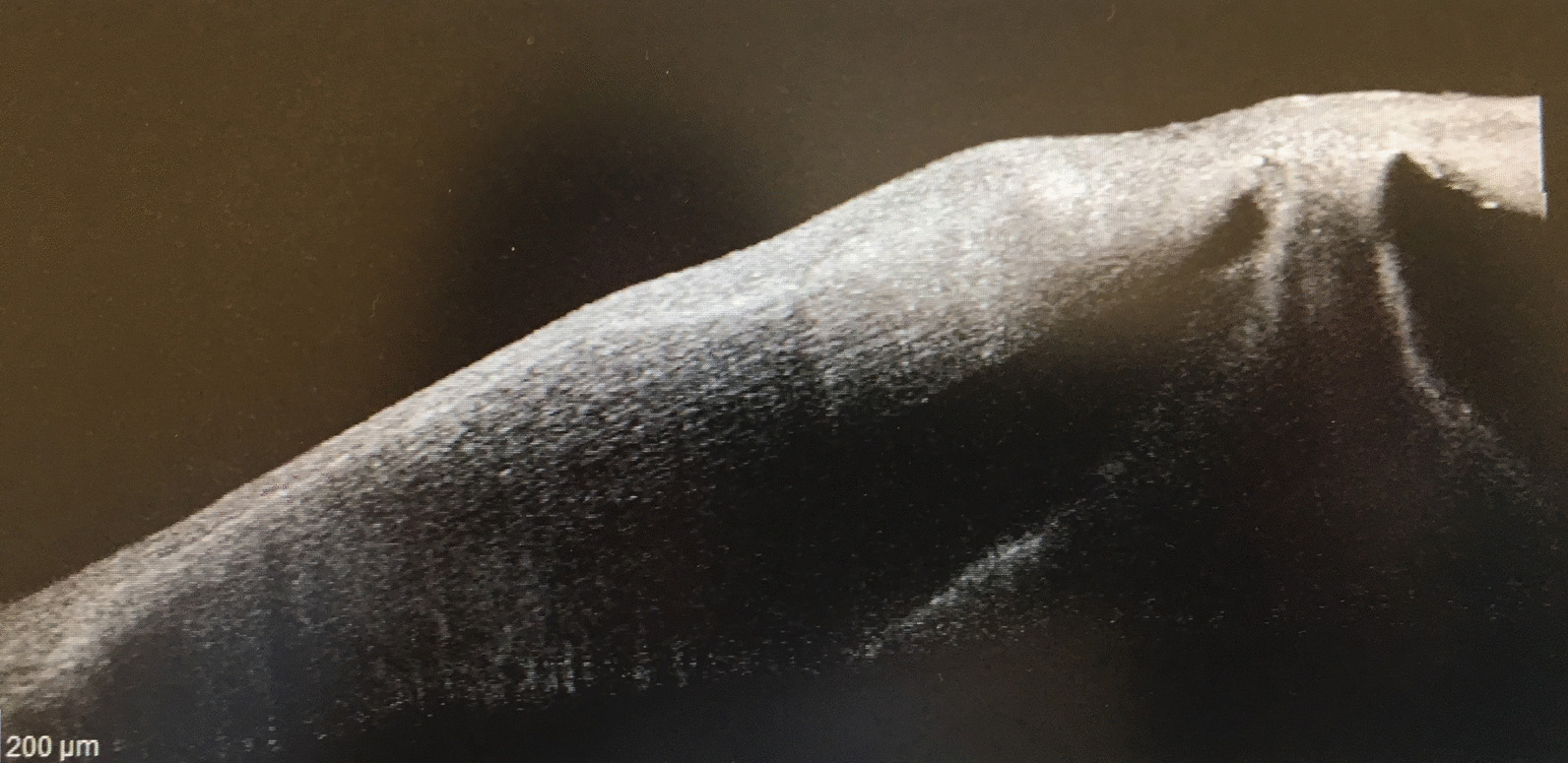
Anterior segment optical coherence tomography (Heidelberg Spectralis): corneal thinning (minimum corneal thickness 101 µm), iris plugging the perforation site

Upon ophthalmic examination, visual acuity (VA) was 20/32 in the oculus dexter (OD) and 20/630 in the OS. Slit lamp examination revealed subtle conjunctival congestion with fine stromal opacities in the OD, and in the OS, moderate ciliary injection, a perforated paracentral corneal ulceration approximately 1 mm in diameter, with a dense perilesional infiltrate, stromal melting, and a few corneal new vessels and stromal opacities around it (Fig. 1). The pupil was peaked but reactive and the iris was plugging the perforation. A shallow, almost flat anterior chamber with clear aqueous humor and a clear lens were found (Fig. 1). No abnormality was noted in the fundus examination of the eyes. Anterior segment optical coherence tomography (OCT, Heidelberg Spectralis) revealed the inclavated iris in the ulceration zone and a thin cornea in the OS and an imminent 100 µm minimum corneal thickness at the site of perforation (Fig. 2).

Ophthalmological evaluation led us to the diagnosis of paracentral corneal perforation.

Systemic treatment with Ceftriaxone was initiated, 1 g every 12 hours, and topical medication was modified to tobramycin hourly, mydriasis with tropicamide and phenylephrine five times a day, lubricants, and occlusive patch.

Evolution under treatment was stationary without signs of improvement. Therefore, 5 days after admission, cyclosporine 1 mg/ml was added to the topical treatment and it was administered 1 drop a day in the evening. Treatment with Methotrexate 7.5 mg/week was continued.

The danger of this sight-threatening situation required rheumatologist expertise. Taking into account the lack of general symptoms and the normal values of C reactive protein and ESR, neither pulse therapy with methylprednisolone, nor cyclophosphamide addition were taken into consideration.

**Fig. 3.**
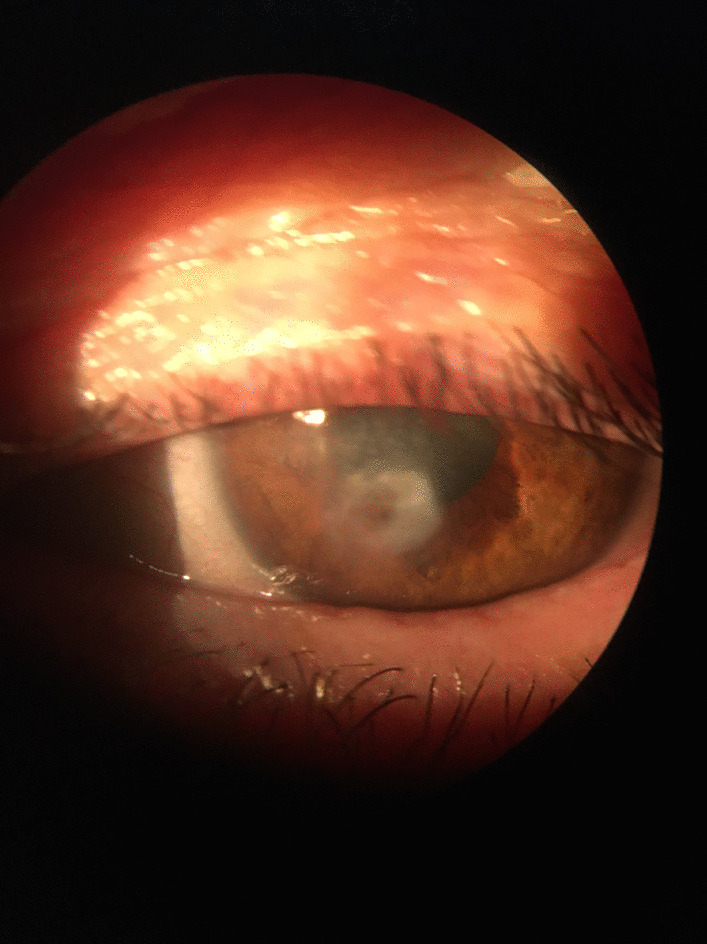
Slit lamp examination of the left eye 7 days following treatment: corneal perforation sealed, dense perilesional infiltrate, and stromal melting diminished considerably, anterior chamber has normal depth

Fortunately, visual acuity started to improve to 20/63, the perilesional infiltrate decreased in size and depth, the iris was liberated from the perforation site and the corneal transparency improved (Fig. 3). At discharge, VA in the OS was 20/25 and the patient was free of symptoms.

**Fig. 4.**
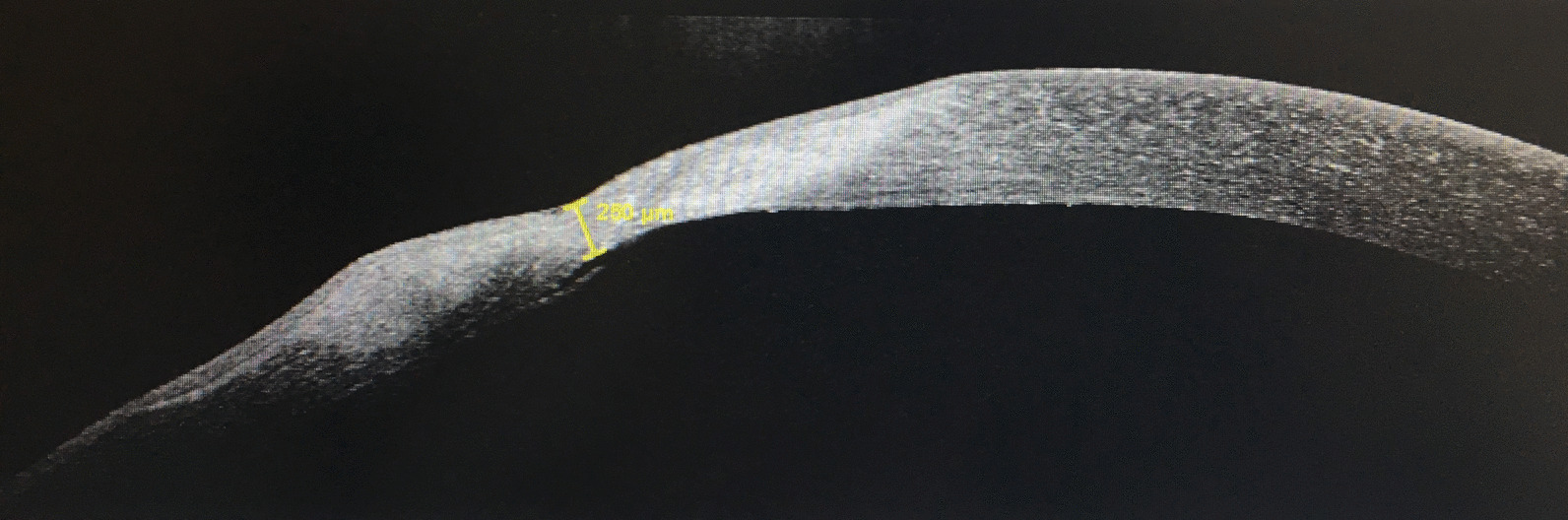
Anterior segment optical coherence tomography of the left eye 7 days after treatment (Heidelberg Spectralis): minimum corneal thickness of 250 µm, iris liberated from the perforation site

Anterior segment optical coherence tomography showed an increase in minimum corneal thickness to 250 µm (Fig. 4). We performed a Schirmer test without anesthesia in the OD, and the result was 1 mm at 5 minutes.

After having been discharged, the patient continued topical treatment with tobramycin three times/day and cyclosporine 1mg/ml, one drop/day every evening and preservative-free eye lubricants; 6 months after this episode, the patient experienced no further ocular complains, her vision is 20/20 in both eyes, she is under rheumatologic and ophthalmological supervision and keeps using preservative-free ocular lubricants.

## Discussion and conclusions

Rheumatoid arthritis (RA), a chronic systemic autoimmune inflammatory entity, is one of the most common collagen vascular diseases. It affects 3–5% of the adult population, more frequently women than men, with an average age at onset of 35–40 years [1]. RA can affect the eye at multiple levels: anterior segment (keratoconjunctivitis sicca, punctate keratopathy, keratitis, episcleritis, scleritis), extraocular muscles, and posterior segment (choroid, retina, optic nerve) [1]. About 90% of patients with RA suffer from dry eye syndrome, especially keratoconjunctivitis sicca, which seems to be correlated with a disease course longer than 10 years [2]. With regard to corneal involvement, many types of keratitis have been described in RA, including peripheral limbal furrow, peripheral or paracentral ulcerative keratitis, keratolysis, acute stromal keratitis, and sclerosing keratitis.

We bring into discussion a case of perforated corneal ulceration in a patient with RA that had been treated for 3 weeks with topical indomethacin, which we managed to treat satisfactorily using a medical approach. Establishing the etiological diagnosis was challenging in this case, and most likely there are several implicating factors: RA definitely plays the main role through modification of the corneal structure and the associated ocular dryness, but the prolonged treatment with topical indomethacin cannot be ruled out, at least at the secondary level.

The pathogenesis of RA-associated corneal ulceration is far from fully elucidated [1]. It seems to be an imbalance between matrix metalloproteinases (MMP) such as MMP-2 in the corneal stroma and MMP-9 in the lacrimal glands and tissue inhibitors of matrix metalloproteinases (TIMP-1) [1, 2, 4]. Reduced levels of TIMP-1 are responsible for high collagenase activity that leads to a keratolytic sterile process [2]. A resulting altered epithelial barrier facilitates the entrance of inflammatory mediators such as monocytes and macrophages in the stroma that subsequently leads to activation of T cells, resulting in production of antibodies and formation of immune complexes. There seems to be significant human leukocyte antigen–DR isotype (HLA-DR) expression by stromal keratocytes and epithelium determined by interferon gamma released by TH2 lymphocytes [1]. Local cytokines such as interleukin-1 and tumor necrosis factor-alpha induce production of collagenase and protease [1, 2, 5, 6]. Paracentral ulcerative keratitis develops in patients with severe dry eye but without marked conjunctival inflammation, such as our case [1, 2].

The initiation of topical cyclosporine therapy was the key to successful treatment given that it arrested the keratolysis and led to ulcer re-epithelialization [7]. There are several reports that recommend topical cyclosporine for corneal ulcer associated with RA [7, 8], as it may enable epithelial healing while reducing cell-mediated immune reactions in the cornea [9]. In our case, topical cyclosporine was initiated with a 5-day delay, as it is expensive and not immediately available. Before the administration of topical cyclosporine, the corneal ulcer did not show significant improvement. Regarding systemic chemotherapy in rheumatoid corneal perforations, there are no clear guidelines, but a strong collaboration between ophthalmologists and rheumatologists is essential to assess the systemic disease status and to determine the need to change or adjust the immunosuppressive therapy.

Although the patient was on immunosuppressive treatment and free of systemic symptoms, she still developed corneal ulceration, highlighting the fact that there are several events initiating corneal lesions and others perpetuating them, such as infection that accelerates corneal melting [5].

The previously diagnosed dry eye syndrome in the context of RA, even under treatment with preservative-free lubricants, favored the ulceration development [8]. There are studies showing that the corneal central thickness and stromal thickness in patients with RA are statistically significantly lower than in controls [1, 2]. Additionally, there is evidence of proteolytic degradation in both corneas of patients ranging from early xerophthalmia to ulcerating xerophthalmia [1, 8].

The use of topical indomethacin in our case is questionable [4, 10]. There have been case reports with patients taking topical NSAIDs who developed corneal perforation that healed after discontinuation [3, 4]. Moreover, it has been shown that topical NSAIDs are associated with corneal hypoesthesia and may be responsible for corneal perforation [3, 4].

Even if the clinical aspect was suggestive of sterile ulcerative keratitis (clear cornea and aqueous), treating the condition exclusively as an autoimmune corneal melt without antibiotic coverage could have resulted in a potentially blinding condition [5]. For logistical reasons we did not have the ability to perform corneal scraping, and therefore we chose topical and systemic treatment with broad-spectrum antibiotics.

Considering the positive outcome following topical cyclosporine, the role of inflammation proved to be essential.

In cases with perforation, the application of cyanoacrylate adhesive, lamellar grafting, or tarsorrhaphy is indicated, depending on ulceration size [1]. In our case, the presence of the iris at the ulceration site acted like a self-adhesive substance.

Nevertheless, photographs of the lesion were helpful in evaluating regression. Anterior segment OCT images were particularly important, highlighting the anatomical improvement.

Corneal perforated ulceration is a rare complication secondary to RA. This case signals and demonstrates that even though the underlying disease was well controlled and monitored, and the dry eye was properly addressed, corneal ulceration still occurred in this RA patient. Indomethacin administered topically may have precipitated corneal perforation, but cyclosporine associated with antibiotics led to good visual outcome. However, since the patient had already been diagnosed with corneal ulcer in another service, there is a possibility that the corneal perforation was a natural course of peripheral ulcerative keratitis (PUK) and undertreatment, rather than due to indomethacin drops. Establishing a cause-effect relationship in this scenario when natural course of the disease is a major confounding factor is difficult and the assumption that indomethacin is the sole culprit here is questionable. Methotrexate 7.5 mg/week is the usual lowest prescribed dose in PUK. Therefore, we can assume that the progression of the ulcer might have been prevented by increasing the methotrexate dose or by adding systemic steroids.

Anterior segment OCT has demonstrated its valuable role in monitoring the disease from a structural perspective.

The main “takeaway” lessons from this case are that topical indomethacin should not be used in corneal ulcers, and that topical cyclosporine was efficacious in healing corneal perforation in a patient with RA.

## Data Availability

The datasets used and analyzed during the current case report are available from the corresponding author on reasonable request.

## Abbreviations

ESR: Erythrocyte sedimentation rate
HLA: Human leukocyte antigen
MMP: Matrix metalloproteinases
NSAIDs: Nonsteroidal anti-inflammatory drugs
OCT: Optical coherence tomography
OD: Right eye (oculus dexter)
OS: Left eye (oculus sinister)
RA: Rheumatoid arthritis
TIMP: Tissue inhibitors of matrix metalloproteinases
VA: Visual acuity
